# A study on Chinese consumer preferences for food traceability information using best-worst scaling

**DOI:** 10.1371/journal.pone.0206793

**Published:** 2018-11-02

**Authors:** Cheng Liu, Jiaoyuan Li, William Steele, Xiangming Fang

**Affiliations:** 1 College of Economics and Management, China Agricultural University, Beijing, China; 2 Department of Agricultural & Applied Economics, University of Georgia, Athens, Georgia, United States of America; 3 School of Public Health, Georgia State University, Atlanta, Georgia, United States of America; Sam Houston State University, UNITED STATES

## Abstract

Food safety is a global public health issue, which often arises from asymmetric information between consumers and suppliers. With the development of information technology in human life, building a food traceability information sharing platform is viewed as one of the best ways to overcome the trust crisis and resolve the problem of information asymmetry in China. However, among the myriad information available from the food supply chain, there is a lack of knowledge on consumer preference. Based on the best-worst scaling approach, this paper investigated consumer preferences for vegetable, pork, and dairy product traceability information. Specifically, this paper measured the relative importance that consumers place on the traceable information. The results indicate that consumers have varying priorities for information in different cases. “Pesticide/veterinary use,” “picking/slaughtering date,” and “fertilizer/feed use” are the most preferred traceable information for Chinese consumers in the case of vegetables, while “picking/slaughtering date” and “history of illness and taking protective measures” are the most preferred information in the case of pork. In the case of dairy products, consumers prefer “processing information,” “environmental information of the origin,” and “traceable tag certification information” most. The results of this study call for the direct involvement of the Chinese government in the food safety information sharing system as following. First, given consumers’ diverse preferences, different types of traceable information should be recorded into the information sharing platform depending on food types. Second, the government could promote the step-by-step construction of such a platform based on the priority of consumers’ preferences. Third, new technology should be applied to guarantee the reliability of traceable information. Finally, local preferences in terms of the way consumers receive and understand information should be taken into consideration.

## Introduction

Being challenged nowadays by the global dimensions of food supply chains, food safety issues are an increasingly essential public health issue worldwide. Unsafe food poses global health threats, endangering everyone [1]. In 2015, the World Health Organization (WHO) reported that an estimated 600 million—almost 1 in 10 people in the world—fall ill every year after eating contaminated food and 420,000 die every year, resulting in the loss of 33 million healthy life years (DALYs). Although the full health effects and economic costs of unsafe food are not known, the global impact on health, trade, and development is considered enormous [2,3]. Concerns about food safety have skyrocketed worldwide. Since 2003, in China, there have been a string of incidents involving food poisonings, discovery of dangerous dyes and additives in food products, fraudulent products, and the sale of food beyond its expiration date, which has caused food safety to become an issue of immense public concern [4]. Alcorn and Ouyang stated that Chinese people reported food-borne disease as the second greatest risk they faced in daily life (just after earthquakes), and 92% of respondents expected themselves to soon become a victim of food poisoning [5].

Food markets in developing countries, which are markets with a lemons problem, are characterized by limited information and an absence of regulation [6,7]. An improved traceability system that can organize information transmission throughout an entire supply chain would be an effective tool to ensure food quality [8]. Such information sharing systems can not only provide consumer safety information, which is helpful in resolving information asymmetry and restoring consumer confidence, but is also useful for both industry and regulators in monitoring food production and distribution, identifying food safety problems, and recalling defective food products. The Chinese government is considering building such a food safety information sharing platform based on the meat and vegetable circulation traceability systems used in several experimental cities in order to resolve the problem of information asymmetry. However, building such food traceability systems with greater amounts of information is expensive and complex, which could lead to financial problems for the information providers [9]. Thus, it is very important to identify Chinese consumer preferences on what types of traceable information that they are interested in and their information priorities.

There are already many studies about consumer preferences for food safety related information, but these vary by food type and region. For example, Ortega et al. [10] assessed Chinese consumer preferences for pork safety attributes and concluded that Chinese consumers had the highest willingness-to-pay (WTP) for government certification, followed by third-party certification, traceability information, and product information labels. However, in the case of infant milk formula, Yin et al. reported that WTP for traceability information was highest for Chinese consumers, followed by brand, country of origin, and place of sale [11]. Similarly, consumers in different countries/regions may have different preferences for the same food type. For example, Loureiro and Umberger [12] analyzed American consumers’ willingness-to-pay for beef with different food safety properties and found that certification by government is more important than any other attribute including country-of-origin labeling and traceability. While for Spanish consumers, the origin of the beef is the most important attribute, followed by quality labeling, production system, and price [13]. Ehmke et al. [14] compared the relative importance of Genetic Modification (GM) use, pesticide use, and origin in the case of white onions among consumers in China, France, Niger, and the United States and found that Chinese consumers are the only subjects who value knowing about pesticide use more than GM use or origin.

As food traceability is considered as an indispensable feature of food safety, numerous studies have been performed on consumer preferences for food traceability information [6,15,16]. However, most of them only treated traceability information as one of the attributes of food safety, rather than examining which types of traceable information with which consumers are most interested. Similar to international literature, there has been limited research on identifying the specific types of traceable information in which Chinese consumers are interested. A study conducted by Jin et al. asked consumers to rank their preferences for eight different types of food traceability information for Apple and found that chemical fertilizers/pesticides used, harvest date, and production standard are the top three most preferred traceability attributes for consumers [17]. Although their study intended to assess consumer preferences for different types of traceable information, some other food safety and quality characteristics such as nutrition and quality certification were included in their list. Furthermore, no studies have ever used the same list of traceability attributes to compare consumer preferences for food traceability information by food type. As China is still at the beginning stage of food traceability implementation, it would be important to understand the relative importance of various traceability attributes for different food types. Based on previous research [6,11,16–18] and the “National Meat and Vegetable Distribution Traceability System Specification (Trial)” released in 2010 by the Chinese Ministry of Commerce and Ministry of Finance, 11 attributes of traceable information were extracted and used in this study to measure their relative importance for three different types of food. Published studies use a range of methods to reveal preferences for traceable information, such as rankings or ratings [17,18], and discrete choice experiments [12,19]. Both rating and ranking are quite simple and common ways to reveal consumer preferences. Even though they may suffer from several types of response biases, including social desirability bias, acquiescence bias, and extreme response bias, they can lead to useful information on respondent preferences through the use of multivariate statistical analysis methods [20,21]. However, ranking or rating methods can only reveal the order of importance, rather than the magnitude of the importance.

Discrete choice experiments (DCEs) are increasingly used in food safety information research. DCE is a powerful method, which closely simulates real-world purchasing decisions by assessing the utility of attributes in various combinations. Researchers are cautioned against eliciting preference information for a large number of attributes (usually not exceeding six attributes) when using the DCE methodology [22,23]. Another limitation of the DCE methodology is the difficulty of interpreting the data, which includes the inability to compare utilities across different experiments [24]. Best-worst scaling (BWS), also known as maximum difference scaling, always generates discriminating results as respondents are asked to choose the BEST and WORST option, and it allows us to get both the order and the strength of importance for all items, which rating or ranking cannot achieve. What is more, although BWS cannot get the WTP for each item like DCE does, BWS could handle a long list of items in a relatively simple method. Since our study elicits preference information for 11 items, we used the best-worst scaling approach to estimate urban Chinese consumers’ preferences on 11 types of food traceable information for vegetable, pork, and dairy products.

## Materials and methods

Our objective was to identify which traceable information, for different types of food, consumers would prefer to know in China. To do this, we used the best-worst scaling object case method to reveal the magnitude of importance for each type of food traceability information in terms of different foods categories (vegetable, pork, and dairy products). Here we chose pork as a representative for meat for two reasons. On the one hand, among all types of meat, pork is the most favored animal protein in China. China is also a large producer of pork. According to the statistics released by the China Statistics Bureau in 2016, pork production in China is about 52.99 million tons, which accounted for approximately 62% of the total domestic production of meat and 48% of the total production in the world. On the other hand, in China, pork is a food that frequently suffers from food safety problems, such as the Shuanghui clenbuterol incident in 2011, excessive antibiotic residues in 2012, the dumping of dead pigs into the Huangpu River in Shanghai in 2013, and the selling of pork from diseased or dead pigs, or “zombie meat,” which has been occurring since 2014.

### Development of BWS

BWS development began with a review of prior literature on food safety issues, especially in China [6,11,16–18]. Table 1 shows the final traceable information chosen: (1) picking/slaughtering date, (2) pesticide/veterinary use, (3) fertilizer/feed use, (4) history of illness and taking protective measures, (5) processing information, (6) packaging information, (7) transportation information, (8) retail information, (9) environmental information of the origin, (10) producers’ information, and (11) traceable tag certification information.

**Table 1:** Attributes & explanation.

Object code	Object names	Object explanation
1	picking/slaughtering date	vegetable pick date; pork slaughter date; raw milk date
2	pesticide/veterinary use	specific information on pesticide/preventative medicine use
3	fertilizer/feed use	specific information on fertilizer/feed use
4	history of illness and taking protective measures	history of illness and protective measures taken
5	processing information	cleaning, sorting, grading information for vegetables; cleaning, segmentation, grading, storage information for pork; dairy products processing factory information, information on relevant staff, quality information for raw milk (test before process), production technical process, information on additives, quality control standards
6	packaging information	specific information on products packaging, such as information on packaging company, quality information (test when packaging)
7	transportation information	specific information on food transportation, such as vehicle information, information on the transporter, vehicle quality control information, time information, transportation track information
8	retail information	retailer information, storage information, sales flow information for the food, current product condition
9	environmental information of the origin	farm environment for vegetable, pork, and raw milk production
10	producers’ information	specific farming information covers vegetable/pig; specific farming information covers dairy products
11	traceable tag certification information	whether the products carried a certification label, and information on the testing institute.

### BWS designs

Generating the choice sets is a key stage in implementing a BWS study, in which an optimal experimental design is needed to meet three criteria as follows [25,26]. First, each item has to appear an equal number of times across all choice sets, which means each kind of traceable information should have the same chance of being selected; Otherwise, one type of traceable information might be over-represented in the choice sets resulting in bias [26,27,28]. Second, each type of information has to co-occur the same number of times with every other information item to control the potential “context effects” [27,28]. This is important because BWS is an attribute-based methodology in which respondents are required to choose the maximally different pair across a number of repeated choice sets [26]. Third, the number of attributes that appear in each choice set must be fixed, usually at three to seven, or there may be “demand effects” [29]. As a result of unequal set sizes, respondents may receive the unintentional signal that they should choose what is expected or desired by the researchers.

**Fig 1:**
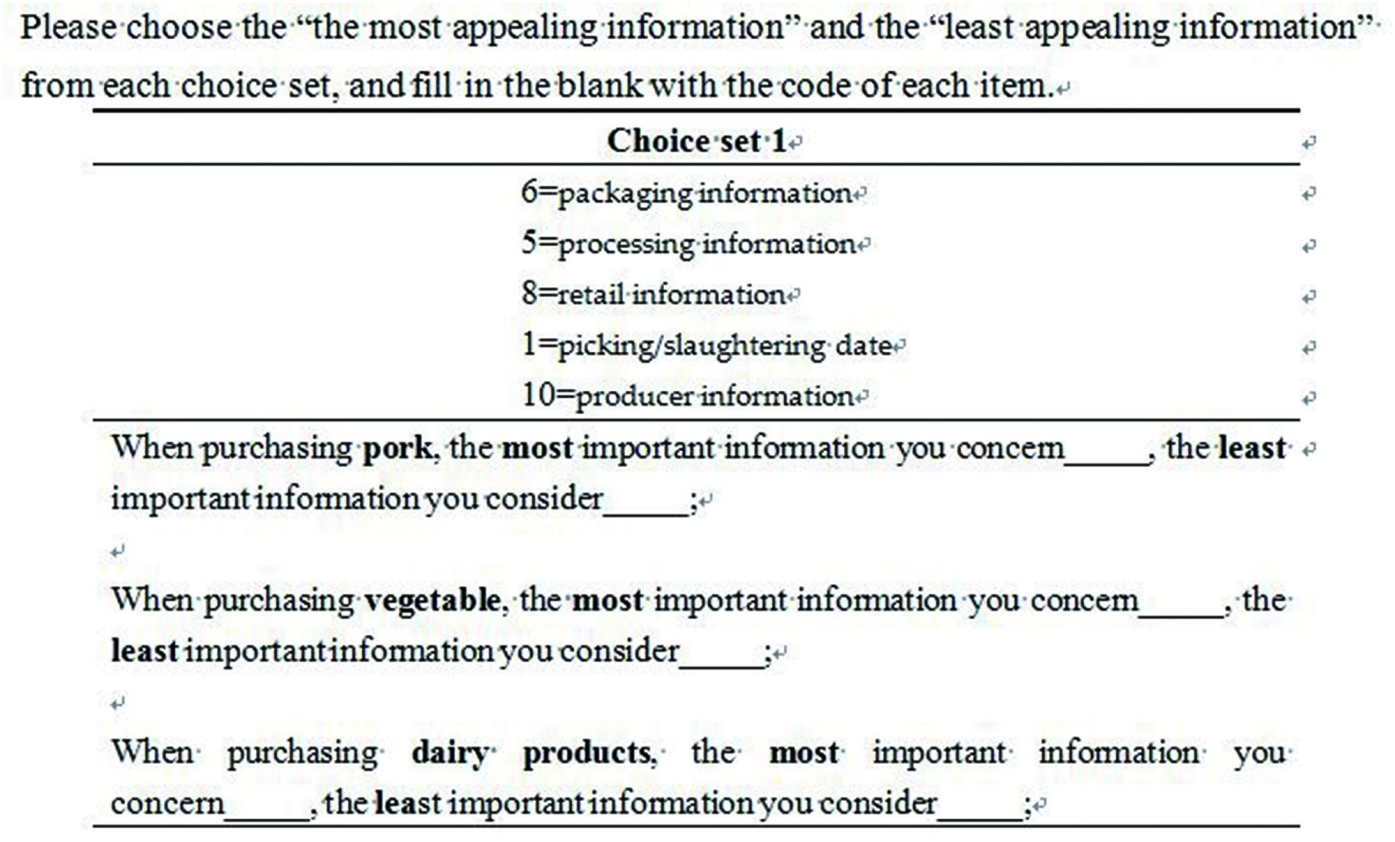
A choice task shown to participants during the paper-based presentation of the best-worst scaling survey. The presentation was conducted in Chinese.

The commonly used standard design in BWS surveys is a balanced incomplete block design (BIBD) design. The main advantage of adopting a BIBD design is that it not only satisfies the three important criteria above, but can also largely reduce the number of comparison sets to be evaluated [26,30]. Given a set of *k* attributes, a BIBD design will create *s* choice sets with *m* attributes in each choice set, which implies that the number *k* is always larger than the number *m*. We then assume that each attribute appears *r* times, and each pair of attributes occurs *λ* times. A BIBD design has the following characteristics: (1) Each attribute occurs in each comparison set at most once, and (2) each of the *m* attributes appears exactly *r* times across the choice sets and co-occurs exactly *λ* times with other (*m −* 1) attributes [31]. Note, numbers *λ* and *r* are integers, and *λ* can be calculated according to the equation of *λ = r* × (*m −* 1) / (*k −* 1). An ideally preferred BIBD design is symmetrical (when *s* = *k*), which is also known as a Youden square [26]. With 11 attributes to be evaluated, SAS software was used to generate a Youden BIBD design, which consists of 11 tasks comparing subsets of the five attributes being examined. Under these experimental conditions, each attribute appeared five times and was presented with each of the other 10 attributes exactly twice. Fig 1 illustrates a typical scenario from the BWS task.

### Sample/Participants

In 2016, surveys were administered in three Chinese cities (Hangzhou in Zhejiang Province, Shijia Zhuang in Hebei Province, and Linyi in Shandong Province) by trained research assistants. These three cities were designated by the Chinese Ministry of Commerce as pilot cities for constructing a meat and vegetable traceability system. This traceable information sharing platform is highly likely to be adopted by the local government in these cities. The three cities are located in the eastern, south central, and north central regions of China, and each have different levels of economic development, living habits, and culture. Thus, the findings based on the survey data collected from these three cities may be able to capture the regional diversity of consumer preferences.

The whole survey procedure, including the procedure for obtaining informed consent, was conducted in the participants’ native language- Chinese under the approvement by the China Agricultural University institutional review board. Before the survey, all participants were both verbally and in writing informed that their participation was completely based on free will, and that the data were collected anonymously on paper questionnaires. Verbal consent was provided by all the respondents at the beginning of the survey. Respondents were asked to finish three tasks for the three types of foods (pork, vegetables, and dairy), respectively. In each task for a certain food, respondents were showed a sequence of 11 different scenarios, one at a time, and asked to choose the best and worst attribute (information) from each of the five attributes (information) within each task. All items were read aloud to participants to ensure that persons of varying degrees of literacy comprehended the material.

To maximize variation, a purposive sample that included people with variations in socio-demographic characteristics (i.e., age, gender, educational background, and social class) was recruited from one farmer’s market, one domestic supermarket, and one local butcher shop in each of the three cities. In all, a total of 108 consumers were interviewed in three cities, with 36 consumers in each city. Three, six, and nine respondents were excluded respectively for pork, vegetables, and dairy because they were missing information on major variables, resulting in a final study sample of 105 participants for pork, 102 for vegetables, and 99 for dairy products. The statistical sample size of consumers obtained in the pork, vegetable, and dairy traceable information survey is 1,155 (105 valid participants×11 choice sets), 1,122 (102 valid participants×11 choice sets), and 1,089 (99 valid participants×11 choice sets), respectively.

### Data analysis

BWS assumes that respondents can easily make reliable and valid choices as they are required to choose the “best” and “worst” options in each choice set. By repeatedly asking them to choose the two most extreme attributes from a series of choice sets, we can tell the level of importance of each attribute. This is determined by the number of times an attribute is chosen as the “best” and “worst” option, the number of respondents, and the number of times that each item occurs in the choice sets. Hence, the level of importance of a particular attribute can be calculated by the following equation:

$$Std.Score=\frac{\text{Best-worst}\;\text{score}}{r\times n}=\frac{Count_{best}-Count_{worst}}{r\times n}$$ (1)

where *Count*_*best*_ is equal to the total number of times each attribute was chosen as the “best” (most preferred), *Count*_*worst*_ is equal to the total number of times each attribute was chosen as the “worst” (least preferred), the best-worst score measures the difference between *Count*_*best*_ and *Count*_*worst*_, and *r* is the times each type of information appears (in this case *r* = 5). *n* is the number of observations.

## Results

### Participants

A total of 102 respondents completed the vegetable survey, 105 respondents completed the pork survey, and 99 respondents completed the dairy survey between June and July 2016. For the vegetable survey, the ages ranged from 15 to 62, with a mean of 32.9 ± 9.9. Of the 102 participants, 60 (58.8%) were men and 42 (41.2%) were women. More than half (59.8%) were married, and 47 (46.1%) had a tertiary education level or above. Only 18 (17.6%) of the respondents’ jobs were related to the food industry. For the pork survey, the average age of the respondents was 32.7 ± 9.9. Of the 105 participants, 61 (58.1%) were men and 44 (41.9%) were women. More than half (59.0%) were married, and 48 (45.7%) had a tertiary education level or above. For the dairy survey, the average age of the respondents was 33.0 ± 9.9. Of the 99 participants, 58 (58.6%) were men and 41 (41.4%) were women. More than half (59.6%) were married, and 45 (45.5%) had a tertiary education level or above. Almost half (over 48% in all three surveys) of the participants were the family food buyer. Characteristics of study participants are shown in Table 2.

**Table 2:** Characteristics of study participants.

Characteristics	Vegetable survey		Pork survey		Dairy survey	
	Mean/No.	SD/%	Mean/No.	SD/%	Mean/No.	SD/%
Age (years)	32.9	9.9	32.7	9.9	33	9.9
Sex						
male	60	58.8%	61	58.1%	58	58.6%
female	42	41.2%	44	41.9%	41	41.4%
Marital status						
Married	61	59.8%	62	59.0%	59	59.6%
Never married	39	38.2%	41	39.0%	39	35.4%
Widowed	2	2.0%	2	1.9%	1	1.0%
Divorced	0	0.0%	0	0.0%	0	0.0%
Education						
Primary or below	2	1.9%	2	1.9%	2	2.0%
Junior high school	6	5.9%	7	6.7%	6	6.1%
High school	47	46.1%	48	45.7%	46	46.5%
Tertiary	47	46.1%	48	45.7%	45	45.5%
Occupation status						
employed	49	48.0%	49	46.7%	46	46.5%
self-employed	18	17.6%	19	18.1%	18	18.2%
unemployed	0	0.0%	0	0.0%	0	0.0%
retired	4	3.9%	4	3.8%	4	4.0%
migrant workers	10	9.8%	11	10.5%	11	11.1%
students	9	8.8%	10	9.5%	8	8.1%
other	12	11.8%	12	11.4%	12	12.1%
Does occupation relate to food industry	18	17.6%	17	16.2%	16	16.2%
Family food buyer	49	48.0%	51	51.4%	48	48.5%

N = 102, 105, 99.

### Tests of data integrity

According to Eq 1, BWS scores can be calculated by subtracting the number of least preferred traceable information attributes from the number of most preferred on an aggregate level. Thus, the “Best-worst score” should between −*r* × *n* (i.e., −5 × 102 = −510) to +*r* × *n* (i.e., +5 × 102 = +510).

Another test for data integrity is whether the sum of the number of times that each type of traceable information was chosen as the most preferred is equal to the sum of the number of times that each was chosen as the least preferred–see the “Sum” for the columns “Best” (most preferred information) and “Worst” (least preferred information) in Table 3. For the total sample, if there are no missing values, both of the sums should be equal to *s* × *n*. What is more, the sum of the “Best-worst score” across all the items should be zero (see the “Sum” for the “Best–worst score” column in Table 3). This is because each respondent is required to provide only two answers–the best and worst in each choice set. As these relationships are true in all BIBD designed BWS studies, one can use these principles to check for data integrity, which is an additional merit of BWS over methods like rating and ranking [26].

**Table 3:** Subjective priority of traceable information preferred by consumers in the case of vegetables.

Rank	Object names	Best	Worst	Best-worst score	Standard score
1	2 pesticide/veterinary use	279	23	256	0.5020
2	1 picking/slaughtering date	230	19	211	0.4137
3	3 fertilizer/feed use	171	21	150	0.2941
4	9 environmental information of the origin	176	37	139	0.2725
5	11 traceable tag certification information	79	65	14	0.0275
6	4 history of illness and taking protective measures	39	51	-12	-0.0235
7	5 processing information	47	96	-49	-0.0961
8	10 producers’ information	40	184	-144	-0.2824
9	6 packaging information	30	188	-158	-0.3098
10	7 transportation information	17	208	-191	-0.3745
11	8 retail information	14	230	-216	-0.4235
	Sum	1122	1122	0	0

Frequency counts and Standardized Score (n = 102).

In the vegetable survey, the sample size (*n*) is 102, and the number of choice sets (*s*) is 11. Thus, the result of *s* × *n* is 1,122. As expected, the sum of both the most preferred and least preferred information in Table 3 equals 1,122 and the sum of the best–worst scores as well as the standard scores equals zero, thus indicating there were no data processing errors. Similarly, we can conclude there were no data processing errors in the pork and dairy survey.

### Preference

**Fig 2:**
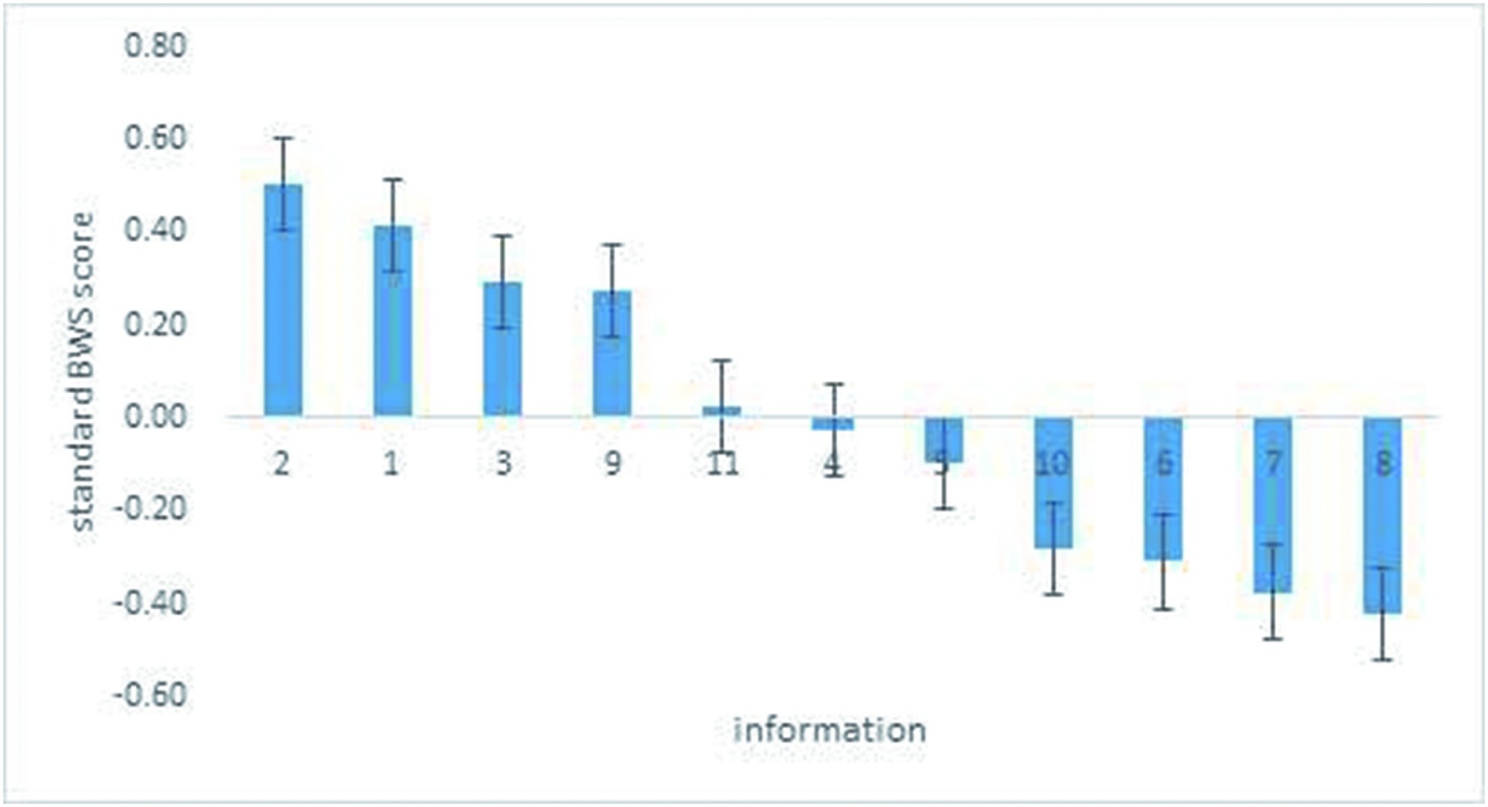
Relative importance of traceable information in the vegetable survey.

The following section shows the results of the BWS reflecting respondents’ trade-off choices as to which kind of traceable information they prefer in terms of different foods (vegetables, pork, dairy). Table 3 shows that the most important information consumers preferred in the vegetable case study is information about “pesticide use” (279 consumers). The second most important attribute is the “picking/slaughtering date” (230 consumers). The least preferred information is retail information (14 consumers). The relative priority assigned to each type of information is represented by the standard scores depicted in Fig 2. Each attribute/information is shown across a horizontal axis, and the standard score is on the vertical axis. The length of the bars represents the relative salience of each kind of information, standardized to a scale ranging from -1.00 to +1.00. All the information receiving a positive score are those above the “0” line, which means those types information are perceived to be preferred. The first four types of information (items 2, 1, 3, and 9) are significantly preferred, and the last four kinds of information (items 10, 6, 7, and 8) are progressively less appealing. “Pesticide use” is 85% more likely than “environmental information” to be chosen as more appealing; “retail information” is 76% more likely than “processing information” to be least appealing. In terms of “history of illness and taking protective measures,” and “processing information,” both are about equal in being neither preferred nor disliked, as their scores is close to “0.”

In the case of pork, people show very similar results to the vegetable survey when choosing the information they dislike, while the results for “best information” are totally different (Table 4). The most important information cited most frequently by respondents is “history of illness and protective measures taken” (255 consumers), followed by “picking/slaughtering date” (254 consumers). “Retail information” and “transportation information” are most frequently cited as being the least important for respondents (221 and 201 consumers, respectively).

**Table 4:** Subjective priority of traceable information preferred by consumers in the case of pork.

Rank	Object names	Best	Worst	Best-worst score	Standard score
1	1 picking/slaughtering date	254	27	227	0.4324
2	4 history of illness and taking protective measures	255	35	220	0.419
3	2 pesticide/veterinary use	191	38	153	0.2914
4	3 fertilizer/feed use	144	36	108	0.2057
5	11 traceable tag certification information	96	72	24	0.0457
6	9 environmental information of the origin	70	60	10	0.019
7	5 processing information	62	78	-16	-0.0305
8	10 producers’ information	38	194	-156	-0.2971
9	6 packaging information	13	193	-180	-0.3429
10	7 transportation information	9	201	-192	-0.3657
11	8 retail information	23	221	-198	-0.3771
	**Sum**	1155	1155	0	0

Frequency counts and Standardized Score (n = 105).

**Fig 3:**
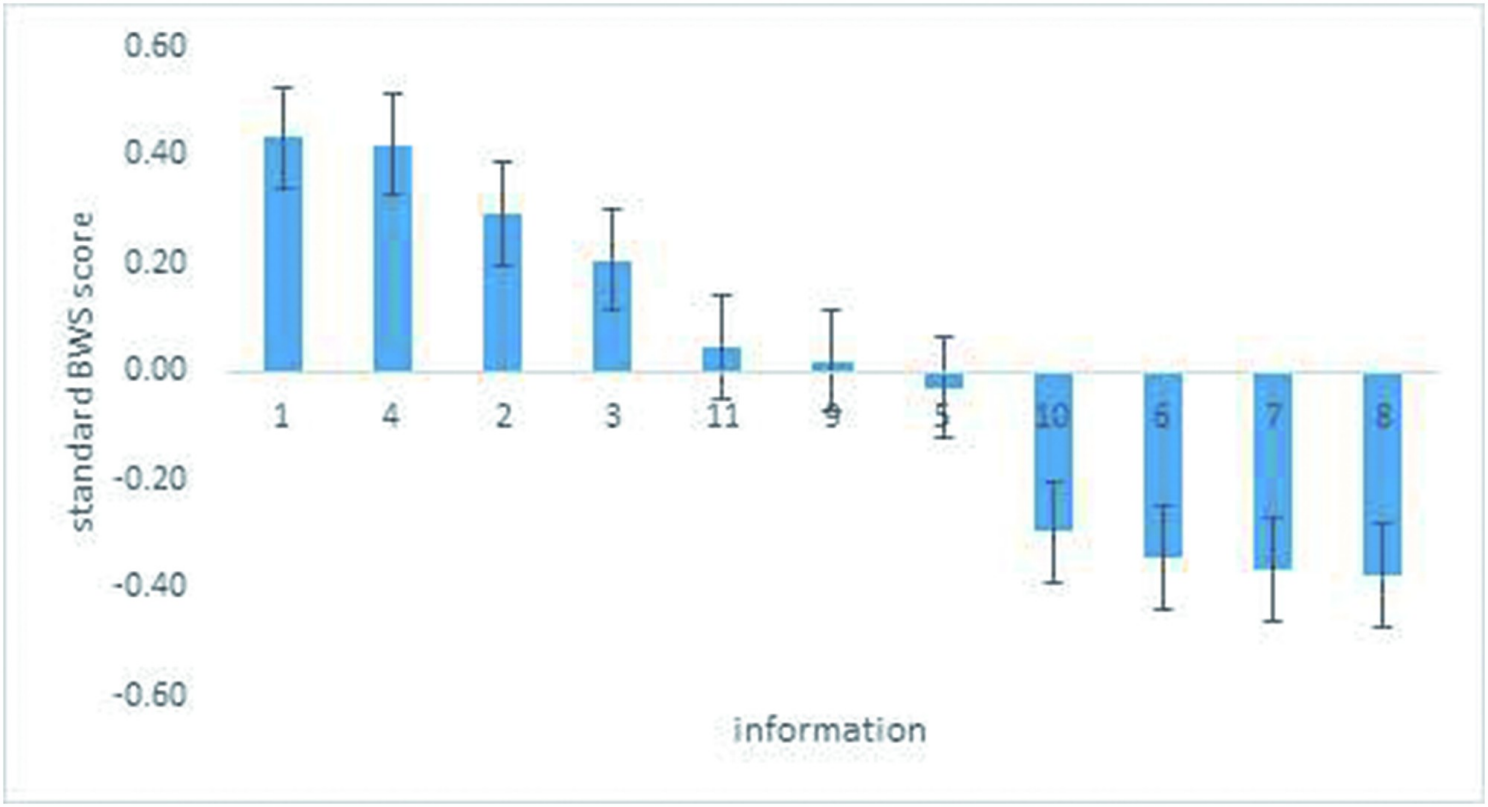
Relative importance of traceable information in the pork survey.

The graphical representation in Fig 3 shows that the first two kinds of information (“picking/slaughtering date,” and “history of illness and taking protective measures”) are preferred about equally (the scores are 0.43 and 0.42, respectively), while the third and the fourth are preferred less. Among the four lesser preferred information, there are no major differences in the standard score; these scores are all strongly less preferred (-0.30, -0.34, -0.37, and -0.37, respectively). “Picking/slaughtering date” is 90.5% more likely than “traceable tag certification information” to be chosen as more important information; “retail information” is only 26.7% more likely than “producers’ information.”

Table 5 presents the results of the data collected using the BWS method in the dairy case study. Unlike vegetables and meat, dairy products have characteristics of both agricultural products and general merchandise at the same time. Based on the results, consumers have different attitudes towards traceable information for dairy products. “Processing information” is the most frequently cited information, followed by “environmental information” and “traceable tag certification” (197, 176, and 151 consumers, respectively). Like the case of pork, respondents most frequently cited the least important information as being “retail information” and “transportation information” (210 and 215 consumers).

**Table 5:** Subjective priority of traceable information preferred by consumers in the case of dairy.

Rank	Object names	Best	Worst	Best-worst score	Standard score
1	5 processing information	197	59	138	0.2788
2	9 environmental information of the origin	176	53	123	0.2485
3	11 traceable tag certification information	151	71	80	0.1616
4	1 picking/slaughtering date	107	28	79	0.1596
5	2 pesticide/veterinary use	95	48	47	0.0949
6	4 history of illness and taking protective measures	91	60	31	0.0626
7	3 fertilizer/feed use	45	46	-1	-0.002
8	6 packaging information	76	145	-69	-0.1394
9	10 producers’ information	74	164	-90	-0.1818
10	8 retail information	43	205	-162	-0.3273
11	7 transportation information	34	210	-176	-0.3556
	**Sum**	1089	1089	0	0

Frequency Counts and Standardized Score (n = 99).

**Fig 4:**
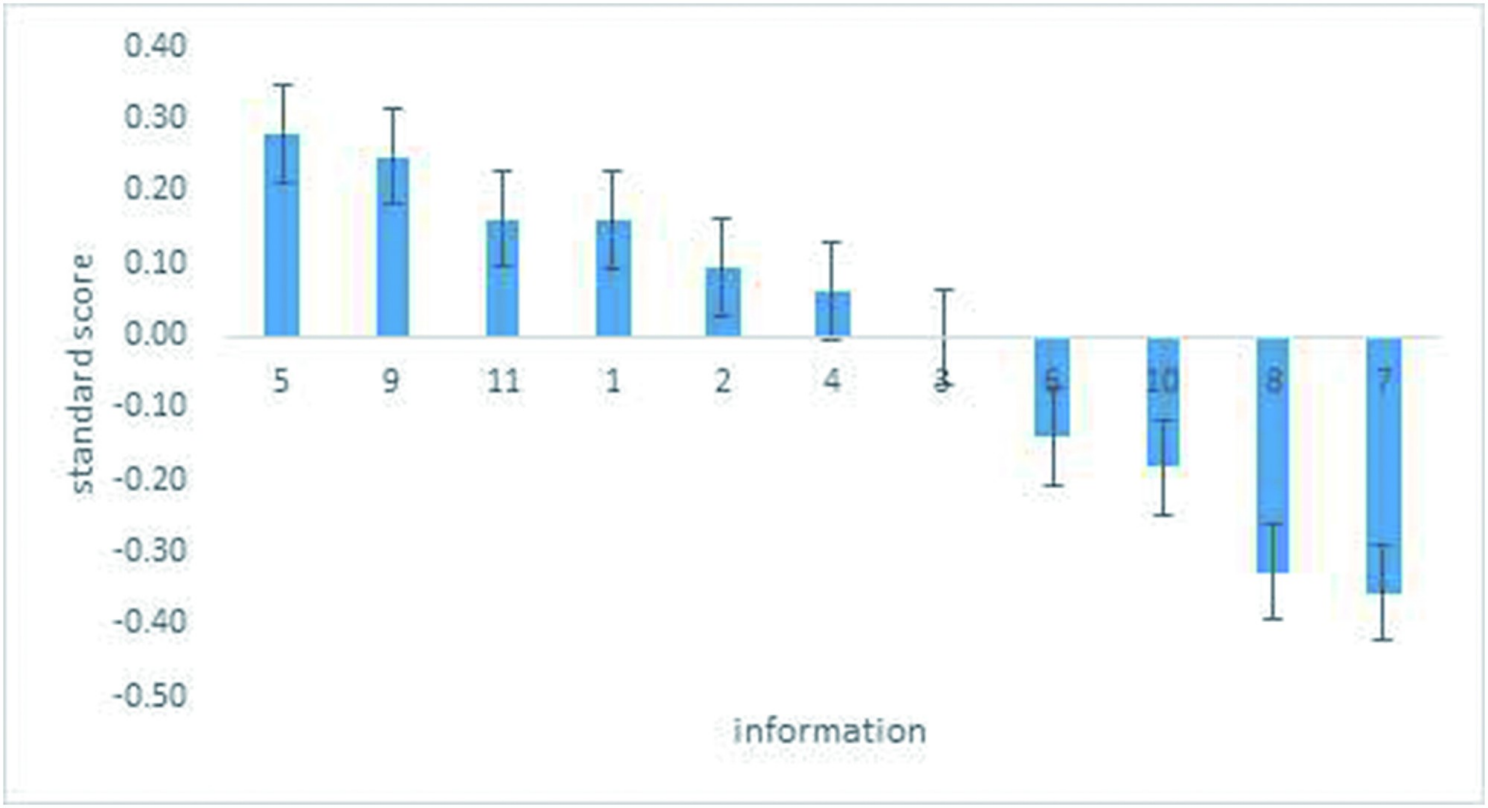
Relative importance of traceable information in the dairy survey.

The results showing the importance of order for each type of information source are presented in Fig 4. Compared to the production of vegetables and pork, dairy products have a more complex production process, so it is reasonable for consumers to choose “processing information” as most important. “Environmental information,” “traceable tag certification information,” and “picking/slaughtering date” follow in importance for consumers. It seems people have a controversial attitude towards “fertilizer/feed use information,” the score for which is about “0.” The last two kinds of information, “transportation information” and “retail information” are strongly less preferred (-0.36 and -0.33).

The graphical representation in Fig 4 also shows the strength of the importance of all kinds of information. “Processing information” is 75% more likely than “traceable tag certification information” and “picking/slaughtering date” to be chosen as more appealing; “transportation information” is 61% more likely than “history of illness and taking protective measures” to be least appealing.

We can see the comparison results in Table 6. For the preferred information, “picking/slaughtering date” is the most appealing information for consumers in all three cases, and the rankings of vegetables, pork, and dairy products are 2, 1, and 4 respectively. “Pesticide/veterinary use” and “environmental information” are also very important to consumers. Consumers show a similar interest in types of traceable information for vegetables and pork, yet the only difference between both cases is that consumers prefer environmental information rather than illness and medical information for vegetables. Results are quite different for dairy products. The most important information for respondents is “processing information,” which is about 12% more likely to be chosen as appealing when compared to “environmental information” and 73% more likely when compared to “traceable tag certification information.”

**Table 6:** Comparison results.

	vegetable		pork		dairy	
	object name	standard score	object name	standard score	object name	standard score
preferred information	2 pesticide/veterinary use	0.5020	1 picking/slaughtering date	0.4324	5 processing information	0.2788
	1 picking/slaughtering date	0.4137	4 history of illness and taking protective measures	0.4190	9 environmental information of the origin	0.2485
	3 fertilizer/feed use	0.2941	2 pesticide/veterinary use	0.2914	11 traceable tag certification information	0.1616
	9 environmental information of the origin	0.2725	3 fertilizer/feed use	0.2057	1 picking/slaughtering date	0.1596
less preferred information	10 producers’ information	-0.2824	10 producers’ information	-0.2971	6 packaging information	-0.1394
	6 packaging information	-0.3098	6 packaging information	-0.3429	10 producers’ information	-0.1818
	7 transportation information	-0.3745	7 transportation information	-0.3657	8 retail information	-0.3273
	8 retail information	-0.4235	8 retail information	-0.3771	7 transportation information	-0.3556

For lesser preferred information, “producers’ information,” “packaging information,” “transportation information,” and “retail information” are cited as the least preferred by consumers. The vegetable and pork surveys show the same order for least preferred information, while the dairy survey does not. For dairy, “packaging information” moves to the top of the less appealing information, which is about 61% less likely to be chosen as the least preferred information than “transportation information” (the overall least preferred). In terms of the magnitude of importance for the types of information, the different types of the food show differing results.

## Discussion and conclusions

In this paper, 11 types of food traceability information were defined for vegetables, pork, and dairy products. On this basis, we applied BWS, a simple but powerful way to analyze consumer preferences on traceable food information in China to be used in the construction of an information sharing platform. Our results show that the preference order for traceable information is quite different depending on the type of food, with the exception of “picking/slaughtering date,” which is the preferred information in all three cases. “Pesticide/veterinary use,” “picking/slaughtering date,” and “fertilizer/feed use” are the most preferred traceable information items for Chinese consumers in the case of vegetables. These findings are similar to those of Jin et al. [17] who also found that chemical fertilizers/pesticides used and harvest date are the most preferred traceability attributes for Apple. This is probably because China ranks as one of the world’s top pesticide and fertilizer consuming countries [32,33]. The increasing overuse of chemical fertilizers and pesticides are posing more and more threats to farmers' health and consumers of agricultural product. “Picking/slaughtering date” and “history of illness and taking protective measures” are the most preferred information items in the case of pork. These findings are consistent with Chen et al. [34] in the sense that Taiwanese consumers also ranked “slaughtering date” as one of the attributes that they were most concerned about. However, measures similar to “history of illness and taking protective measures” were only ranked in the mid-range by Taiwanese consumers [34]. The reason that “history of illness and taking protective measures” was ranked as one of the top two preferred traceable information items in China could be partly due to the continuing incidents of selling pork from diseased or dead pigs, or “zombie meat,” in the past several years. In the case of dairy products, consumers prefer “processing information,” “environmental information of the origin,” and “traceable tag certification information” most. Ranking “processing information” as the most preferred information may signify that the negative impacts of the notorious melamine-contaminated milk event that occurred in 2008 might still remain a major barrier to consumer confidence. The findings that consumer preferences for traceable information vary by food type are similar to the findings of Han et al. [35] in that consumers showed differing concerns about food safety information when purchasing different types of food. Appearing fresh, picking date, fertilizer (agricultural veterinary), and sales reputation are pieces of information with which consumers are most concerned in the case of vegetables; brand, sale information, processing information, and certification will have a greater effect on consumers purchase behaviors in the case of dairy products [35].

It is worth noting that the relative importance of the preferred food traceability information was able to be revealed here via the new method of BWS. The results show a large discrepancy. For vegetables, “pesticide/veterinary use” is about 21% more likely to be cited as the most appealing information when compared to “picking date,” 71% more likely when compared to “fertilizer/feed use,” and 84% more likely when compared to “environmental information.” For pork, “picking/slaughtering date” is only about 3% more likely to be chosen as the most preferred information when compared to “history of illness and taking protective measures,” and it is 48% more likely when compared “pesticide/veterinary use.” For dairy, “processing information” is the most preferred information, which is 12% more likely to be chosen when compared to “environmental information,” and 73% more likely to be chosen when compared to “traceable tag certification information.” The results imply that “pesticide/veterinary use” is the most important information for consumers, and much more important than other information for consumers of vegetables; “picking/slaughtering date” and “history of illness and taking protective measures” are of almost the same important for consumers of pork; and “processing information” is much more important in the case of dairy.

In this study, we identified the main types of information about which consumers are concerned and the relative importance of various types of information, and we found consumers showed varying preferences regarding different food categories. The results of this study should encourage both the Chinese government and industry to present information that meets consumer demands via a traceable information sharing platform that will reduce consumer information asymmetries, increase the reliability of products [34], and lessen the consumer trust crisis [19]. Furthermore, the implementation of a traceable information sharing platform will not only save the time that consumers spend on information seeking [34], but also promote consumer supervision of the food industry. From a market perspective, traceability systems may result in more effective demands, a prevalence of products using this system, and increased profits for firms due to an increased demand for safe products. As a result, market bodies along the production chain can contribute to a larger, safer food market for consumers.

In China, the government is an important driver in the development of a safe food market. Beyond the implementation of a traceable information sharing platform, the conclusions from this research may improve relevant support policies. The following policy implications are proposed.

First, in terms of the diverse consumer preferences for food traceability information depending on food type, different types of traceable information should be recorded when establishing the (national comprehensive) traceable information sharing platform. Policy measures, such as encouraging food firms to adopt traceability system and record food traceability information, should be introduced to promote the construction of a traceable information sharing platform.

Second, because it is time-consuming and costly to construct such a complicated information sharing platform, the government should begin by recording information that is already available based on its relative importance to accommodate budget constraints and to encourage all food production stakeholders to join in on efforts to establish and apply such a platform.

Third, to guarantee the reliability of traceable information recorded and the efficacy of the information sharing platform, policy instruments should be better designed to encourage the adoption of internet technology and Blockchain technology in the food industry as well as promote the application of Internet Technology (IT) and Artificial Intelligence Technology (AIT) to communicate information. Because consumer trust in the information sharing platform is important, once the contents are deemed inaccurate or inadequate, consumer confidence in the effectiveness of traceability is likely to diminish very quickly [15].

Finally, it should be noted that there are inter-regional and individual differences among Chinese consumers. In the development of a traceable information sharing platform, the government should account for local preferences and the differing ways consumers receive and understand traceable information when the platform is designed.

As with any research study, there are several limitations that should be noted. The first limitation in this research was its use of a small sample size, and participants were selected from only three cities. A larger and more representative sample in a future study would be expected to give more reliable results. Second, this study used the BWS (object case) method to measure the relative importance that consumers place on traceable information. However, the BWS (object case) design will not allow us to estimate consumers’ willingness to pay for the traceable information. Based on the results of this study, our next plan is to estimate consumer willingness to pay using the DCE method, which could aid in finding out the economic values of the different types of traceable information, which will better assist future decision making and priority setting. Last, but not least, as the first step of our project, this study aims to understand Chinese consumers’ overall preferences on food traceability information for different food types. Examining the preference heterogeneity among consumers via including more sociodemographic factors (such as age, education, gender) [18] and psychosocial constructs (such as risk perception, trust, and habits) [36,37] in a food traceability system is the next step we will take.

## Supporting information

S1 File Questionnaire used in the study (in Chinese). (PDF)

S2 File Questionnaire used in the study (in English). (PDF)

S3 File Data used in the study. (XLSX)
