# Supplementary material for: A study on Chinese consumer preferences for food traceability information using best-worst scaling
Source: PLoS One. 2018 Nov 2;13(11):e0206793. doi: 10.1371/journal.pone.0206793 (PMC6214548; doi:10.1371/journal.pone.0206793)
Supplement: S1 File — (PDF) [file pone.0206793.s001.pdf]

被调查者代码:

农产品质量安全主题调研

____________省__________市___________区/县

____________街道/乡镇_____________村

具体调查地点： （1）农贸市场

（2）超市

（3）肉制品店

（4）其他

调查员姓名：____________

调查员联系手机：_____________

调查日期：_________________

中国农业大学

2016年6月

问卷说明：本问卷仅用于学术研究，不涉及任何您的个人身份信息。您有权利选择不参与调研，也可以随时退出参与。

A.食品可追溯信息需求

“可追溯信息”是指食品生产、加工、流通和消费各个环节中，与食品质量安全相关的信息。

请在以下各个选项卡所列的信息中挑选出您认为**“最符合您需求”**以及**“最不符合您需求”**的选项，并将**代码**填写在_______上。

**选项卡 1**
6=产品包装信息<br>5=产品加工处理信息<br>8=产品销售信息<br>1=采摘/屠宰日期<br>10=生产者信息
在购买**猪肉**时，对您而言**最需要**的信息是_____，**最不需要**的信息是____；<br>在购买**蔬菜**时，对您而言**最需要**的信息是_____，**最不需要**的信息是____；<br>在购买**乳制品**时，对您而言**最需要**的信息是_____，**最不需要**的信息是____；

**选项卡 2**
9=产地环境信息<br>7=产品物流信息<br>6=产品包装信息<br>8=产品销售信息<br>4=患病史及采取防护措施记录
在购买**猪肉**时，对您而言**最需要**的信息是_____，**最不需要**的信息是____；<br>在购买**蔬菜**时，对您而言**最需要**的信息是_____，**最不需要**的信息是____；<br>在购买**乳制品**时，对您而言**最需要**的信息是_____，**最不需要**的信息是____；

**选项卡 3**
4=患病史及采取防护措施记录<br>10=生产者信息<br>9=产地环境信息<br>2=农药/兽药使用<br>5=产品加工处理信息
在购买**猪肉**时，对您而言**最需要**的信息是_____，**最不需要**的信息是____；<br>在购买**蔬菜**时，对您而言**最需要**的信息是_____，**最不需要**的信息是____；<br>在购买**乳制品**时，对您而言**最需要**的信息是_____，**最不需要**的信息是____；

**选项卡 4**
5=产品加工处理信息<br>3=化肥/饲料使用<br>7=产品物流信息<br>6=产品包装信息<br>2=农药/兽药使用
在购买**猪肉**时，对您而言**最需要**的信息是_____，**最不需要**的信息是____；<br>在购买**蔬菜**时，对您而言**最需要**的信息是_____，**最不需要**的信息是____；<br>在购买**乳制品**时，对您而言**最需要**的信息是_____，**最不需要**的信息是____；

**选项卡 5**
10=生产者信息<br>6=产品包装信息<br>4=患病史及采取防护措施记录<br>11=可追溯标签认证信息<br>3=化肥/饲料使用
在购买**猪肉**时，对您而言**最需要**的信息是_____，**最不需要**的信息是____；<br>在购买**蔬菜**时，对您而言**最需要**的信息是_____，**最不需要**的信息是____；<br>在购买**乳制品**时，对您而言**最需要**的信息是_____，**最不需要**的信息是____；

**选项卡 6**
2=农药/兽药使用<br>1=采摘/屠宰日期<br>11=可追溯标签认证信息<br>9=产地环境信息<br>6=产品包装信息
在购买**猪肉**时，对您而言**最需要**的信息是_____，**最不需要**的信息是____；<br>在购买**蔬菜**时，对您而言**最需要**的信息是_____，**最不需要**的信息是____；<br>在购买**乳制品**时，对您而言**最需要**的信息是_____，**最不需要**的信息是____；

**选项卡 7**
3=化肥/饲料使用<br>2=农药/兽药使用<br>1=采摘/屠宰日期<br>4=患病史及采取防护措施记录<br>8=产品销售信息
在购买**猪肉**时，对您而言**最需要**的信息是_____，**最不需要**的信息是____；<br>在购买**蔬菜**时，对您而言**最需要**的信息是_____，**最不需要**的信息是____；<br>在购买**乳制品**时，对您而言**最需要**的信息是_____，**最不需要**的信息是____；

选项卡 8
7=产品物流信息<br>9=产地环境信息<br>10=生产者信息<br>3=化肥/饲料使用<br>1=采摘/屠宰日期
在购买**猪肉**时，对您而言**最需要**的信息是_____，**最不需要**的信息是____；<br>在购买**蔬菜**时，对您而言**最需要**的信息是_____，**最不需要**的信息是____；<br>在购买**乳制品**时，对您而言**最需要**的信息是_____，**最不需要**的信息是____；

选项卡 9
8=产品销售信息<br>11=可追溯标签认证信息<br>2=农药/兽药使用<br>10=生产者信息<br>7=产品物流信息
在购买**猪肉**时，对您而言**最需要**的信息是_____，**最不需要**的信息是____；<br>在购买**蔬菜**时，对您而言**最需要**的信息是_____，**最不需要**的信息是____；<br>在购买**乳制品**时，对您而言**最需要**的信息是_____，**最不需要**的信息是____；

选项卡 10
1=采摘/屠宰日期<br>4=患病史及采取防护措施记录<br>5=产品加工处理信息<br>7=产品加工处理信息<br>11=可追溯标签认证信息
在购买**猪肉**时，对您而言**最需要**的信息是_____，**最不需要**的信息是____；<br>在购买**蔬菜**时，对您而言**最需要**的信息是_____，**最不需要**的信息是____；<br>在购买**乳制品**时，对您而言**最需要**的信息是_____，**最不需要**的信息是____；

选项卡 11
11=可追溯标签认证信息<br>8=产品销售信息<br>3=化肥/饲料使用<br>5=产品加工处理信息<br>9=产地环境信息
在购买**猪肉**时，对您而言**最需要**的信息是_____，**最不需要**的信息是____；<br>在购买**蔬菜**时，对您而言**最需要**的信息是_____，**最不需要**的信息是____；<br>在购买**乳制品**时，对您而言**最需要**的信息是_____，**最不需要**的信息是____；

B.被访者基本情况

性别	年龄	教育程度<br>(代码 1)	婚姻状况<br>(代码 2)	家庭常住人口数	家庭月收入	个人月收入	职业<br>(代码 3）	职业是否与食品行业相关	是否家庭食品购买决策者
B1	B2	B3	B4	B5	B6	B7	B8	B9	B10

性别：1=男 0=女

教育程度代码：1=小学及以下；3=初中；4=高中；5=本科及以上

婚姻状况：0=未婚 1=已婚 2=离婚 3=丧偶

职业代码：1=被雇佣（含公务员、企事业单位人员）；2=个体和私营劳动者；3=无业者；4=离退休人员；5=外来务工人员；6=学生；7=其他

C.被访者对食品安全情况感知

1.您认为如今食品安全的总体情况如何？_______

1=非常不安全；2=不大安全；3=一般；4=比较安全；5=非常安全

2.您认为如今农产品(生鲜食品)质量安全情况如何？_______

1=非常不安全；2=不大安全；3=一般；4=比较安全；5=非常安全

3.您对食品安全的关注程度如何？_______

1=非常关注；2=比较关注；3=一般；4=不太关注；5=不关注

4.您知道农产品可追溯系统吗？

1=了解；2=听说过但不了解；3=不清楚；4=没听说过

5.您倾向于通过何种方式查询农产品的可追溯信息？

1=超市、农贸市场等的追溯查询机；2=网站输入追溯码；3=手机App扫码查询；4=发短信；5=打电话；6=其他方式（请注明）：______________________
