# Supplementary material for: A study on Chinese consumer preferences for food traceability information using best-worst scaling
Source: PLoS One. 2018 Nov 2;13(11):e0206793. doi: 10.1371/journal.pone.0206793 (PMC6214548; doi:10.1371/journal.pone.0206793)
Supplement: S2 File — (PDF) [file pone.0206793.s002.pdf]

ID:

Research on consumers' preferences for food traceability information

____________Province__________City___________District

Place：

　（1）farmer's market______

　（2）supermarket________

　（3）butcher shop________

　（4）else________________

Investigator Name： ____________

Investigator Phone No.： _____________

Survey date： ________________

China Agricultural University

June 2016

This questionnaire is for academic research only. Your participation is entirely up to you. You may choose not to participate and you are free to withdraw from participation at any time. The data were collected anonymously; all respondents were assigned an ID number prior to data analyses.

A. The preference of traceable information

Food traceable information refers to the safety information related throughout the supply chain, such as the stage of production, processing, circulation, and consumption.

Please choose the "**most appealing information**" and the "**least appealing information**" form each Choice set, and fill in the blank with the code of each item.

**Choice set 1**
6=packaging information<br>5=processing information<br>8=retail information<br>1=picking/slaughtering date<br>10=producers' information
When purchasing **pork**, the **most** information you concern_____, the **least** information you concern____;<br><br>When purchasing **vegetable**, the **most** information you concern_____, the **least** information you concern____;<br><br>When purchasing **dairy products**, the **most** information you concern_____, the **least** information you concern____;

**Choice set 2**
9=environmental information of the origin<br>7=transportation information<br>6=packaging information<br>8=retail information<br>4=history of illness and taking protective measures
When purchasing **pork**, the **most** information you concern_____, the **least** information you concern____;<br><br>When purchasing **vegetable**, the **most** information you concern_____, the **least** information you concern____;<br><br>When purchasing **dairy products**, the **most** information you concern_____, the **least** information you concern____;

Choice set 3
4=history of illness and taking protective measures<br>10=producers' information<br>9=environmental information of the origin<br>2=pesticide/veterinary use<br>5=processing information
When purchasing **pork**, the **most** information you concern_____, the **least** information you concern____;<br><br>When purchasing **vegetable**, the **most** information you concern_____, the **least** information you concern____;<br><br>When purchasing **dairy products**, the **most** information you concern_____, the **least** information you concern____;

Choice set 4
5=processing information<br>3=fertilizer/feed use<br>7=transportation information<br>6=packaging information<br>2=pesticide/veterinary use
When purchasing **pork**, the **most** information you concern_____, the **least** information you concern____;<br><br>When purchasing **vegetable**, the **most** information you concern_____, the **least** information you concern____;<br><br>When purchasing **dairy products**, the **most** information you concern_____, the **least** information you concern____;

Choice set 5
10=producers' information<br>6=packaging information<br>4=history of illness and taking protective measures<br>11=traceable tag certification information<br>3=fertilizer/feed use
When purchasing **pork**, the **most** information you concern_____, the **least** information you concern____;<br><br>When purchasing **vegetable**, the **most** information you concern_____, the **least** information you concern____;<br><br>When purchasing **dairy products**, the **most** information you concern_____, the **least** information you concern____;

Choice set 6
2=pesticide/veterinary use<br>1=picking/slaughtering date<br>11=traceable tag certification information<br>9=environmental information of the origin<br>6=packaging information
When purchasing **pork**, the **most** information you concern_____, the **least** information you concern____;<br><br>When purchasing **vegetable**, the **most** information you concern_____, the **least** information you concern____;<br><br>When purchasing **dairy products**, the **most** information you concern_____, the **least** information you concern____;

Choice set 7
3=fertilizer/feed use<br>2=pesticide/veterinary use<br>1=picking/slaughtering date<br>4=history of illness and taking protective measures<br>8=retail information
When purchasing **pork**, the **most** information you concern_____, the **least** information you concern____;<br><br>When purchasing **vegetable**, the **most** information you concern_____, the **least** information you concern____;<br><br>When purchasing **dairy products**, the **most** information you concern_____, the **least** information you concern____;

Choice set 8
7=transportation information<br>9=environmental information of the origin<br>10=producers' information<br>3=fertilizer/feed use<br>1=picking/slaughtering date
When purchasing **pork**, the **most** information you concern_____, the **least** information you concern____;<br><br>When purchasing **vegetable**, the **most** information you concern_____, the **least** information you concern____;<br><br>When purchasing **dairy products**, the **most** information you concern_____, the **least** information you concern____;

**Choice set 9**
8=retail information<br>11=traceable tag certification information<br>2=pesticide/veterinary use<br>10=producers' information<br>7=transportation information
When purchasing **pork**, the **most** information you concern_____, the **least** information you concern____;<br><br>When purchasing **vegetable**, the **most** information you concern_____, the **least** information you concern____;<br><br>When purchasing **dairy products**, the **most** information you concern_____, the **least** information you concern____;

**Choice set 10**
1=picking/slaughtering date<br>4=history of illness and taking protective measures<br>5=processing information<br>7=transportation information<br>11=traceable tag certification information
When purchasing **pork**, the **most** information you concern_____, the **least** information you concern____;<br><br>When purchasing **vegetable**, the **most** information you concern_____, the **least** information you concern____;<br><br>When purchasing **dairy products**, the **most** information you concern_____, the **least** information you concern____;

**Choice set 11**
11=traceable tag certification information<br>8=retail information<br>3=fertilizer/feed use<br>5=processing information<br>9=environmental information of the origin
When purchasing **pork**, the **most** information you concern_____, the **least** information you concern____;<br><br>When purchasing **vegetable**, the **most** information you concern_____, the **least** information you concern____;<br><br>When purchasing **dairy products**, the **most** information you concern_____, the **least** information you concern____;

B. Information About You

Gender	Age (years)	Education (Code 1)	Marital status (Code2)	Family size	Household income (month)	Personal income (month)	Occupation status (Code3）	Occupation related to food industry or not?	Always the buyer of the family food purchase or not?
B1	B2	B3	B4	B5	B6	B7	B8	B9	B10

Gender: 1=male 0=female

Educaton: 1=Primary or below; 2= Junior high school; 3=High school; 4=Tertiary

Marital status: 1=Married; 2=Never married; 3=Widowed; 4=Divorced

Occupation status: 1=employed; 2=self-employed; 3=unemployed; 4=retired; 5=migrant workers; 6=students; 7=other

C. Attitude toward food safety

1. Please comment on the present situation of the **food safety**. _______

   1=Very unsafe; 2=Unsafe; 3=So-so; 4=Good; 5=Excellent

2. Please comment on the present situation of the **Agri-food safety**. _______

   1=Poor; 2=Unsafe; 3=So-so; 4=Good; 5=Excellent

3. Your **concerning degree** about the food safety.

   1=care for very much; 2=care for much; 3=care for; 4=less care for; 5=never care for

4. Do you know food traceable system?

   1=Yes; 2=Just heard about; 3=Not clear; 4=Never heard

5. Which **approach** do you prefer to get food traceable information?

   1 = Machine in supermarkets or farmer markets, etc; 2= Website; 3= Mobile phone Apps; 4= Text message; 5= Call; 6= Other ways (please specify): ________
